# Antiemetic prophylaxis with droperidol in morphine-based intravenous patient-controlled analgesia: a propensity score matched cohort study

**DOI:** 10.1186/s12871-023-02319-2

**Published:** 2023-10-28

**Authors:** Jia Qi Tan, Hsiang-Ling Wu, Yi-Chien Wang, Juan P. Cata, Jui-Tai Chen, Yih-Giun Cherng, Ying-Hsuan Tai

**Affiliations:** 1 Department of Anesthesiology, Shuang Ho Hospital, Taipei Medical University, 23561 New Taipei City, Taiwan; 2 Department of Anesthesiology, School of Medicine, College of Medicine, Taipei Medical University, 11031 Taipei, Taiwan; 3 Department of Anesthesiology, Taipei Veterans General Hospital, 11217 Taipei, Taiwan; 4 School of Medicine, National Yang Ming Chiao Tung University, 11221 Taipei, Taiwan; 5 Department of Anesthesiology and Perioperative Medicine, The University of Texas MD Anderson Cancer Center, 1515 Holcombe Blvd, Unit 409, 77030 Houston, TX USA

**Keywords:** Opioids, Postoperative nausea and vomiting, Prevention, Prophylaxis, Retching

## Abstract

**Background:**

There are limited real-world data regarding the use of droperidol for antiemetic prophylaxis in intravenous patient-controlled analgesia (IV-PCA). This study aimed to evaluate the antiemetic benefits and sedation effects of droperidol in morphine-based IV-PCA.

**Methods:**

Patients who underwent major surgery and used morphine-based IV-PCA at a medical center from January 2020 to November 2022 were retrospectively analyzed. The primary outcome was the rate of any postoperative nausea and/or vomiting (PONV) within 72 h after surgery. Propensity score matching was used to match patients with and without the addition of droperidol to IV-PCA infusate in a 1:1 ratio. Multivariable conditional logistic regression models were used to calculate adjusted odds ratios (aORs) with 95% confidence intervals (CIs).

**Results:**

After matching, 1,104 subjects were included for analysis. The addition of droperidol to IV-PCA reduced the risk of PONV (aOR: 0.49, 95% CI: 0.35–0.67, *p* < 0.0001). The antiemetic effect of droperidol was significant within 36 h after surgery and attenuated thereafter. Droperidol was significantly associated with a lower risk of antiemetic uses (aOR: 0.58, 95% CI: 0.41–0.80, *p* = 0.0011). The rate of unintentional sedation was comparable between the patients with (9.1%) and without (7.8%; *p* = 0.4481) the addition of droperidol. Postoperative opioid consumption and numeric rating scale acute pain scores were similar between groups.

**Conclusions:**

The addition of droperidol to IV-PCA reduced the risk of PONV without increasing opiate consumption or influencing the level of sedation. However, additional prophylactic therapies are needed to prevent late-onset PONV.

**Supplementary Information:**

The online version contains supplementary material available at 10.1186/s12871-023-02319-2.

## Background

Postoperative nausea and vomiting (PONV) is one of the most common causes of patient distress after surgery, with a reported rate of 20–40% [1, 2]. The pathogenesis of PONV is multifactorial and can be attributed to patient-related (e.g., sex, smoking status, and history of PONV), surgery-related (e.g., type of surgery), and anesthesia-related factors (e.g., use of opioids and volatile anesthetics) [3, 4]. For high-risk patients, the incidence of PONV can be up to 80% [3, 4].

Many surgical patients report that PONV is a worse problem than postoperative pain [5]. Although PONV is usually self-limited, postoperative vomiting or retching can contribute to rare but severe morbidities, such as pulmonary aspiration, wound dehiscence, elevated intracranial pressure, and pneumothorax [6, 7]. In addition, PONV may prolong the length of stay in post-anesthesia care units and cause unanticipated hospitalization following ambulatory surgery [8]. Prophylaxis and treatment for PONV exert a heavy economic burden on healthcare systems [9].

Intravenous patient-controlled analgesia (IV-PCA) is an effective modality to relieve postoperative acute pain. Given that opioids remain the mainstay of analgesics, PONV is a common adverse event during IV-PCA with a reported rate of 18 to 23% [10, 11]. Approximately 12% of surgical patients have been reported to cease IV-PCA early due to intractable PONV [12]. Droperidol is a D2 receptor antagonist that acts centrally on the chemoreceptor trigger zone as an antiemetic agent [13, 14]. The antiemetic efficacy of droperidol was demonstrated in opioid-based IV-PCA. However, previous studies had methodological flaws, including small patient samples (*n* < 1,000) [15–22], insufficient confounding adjustment [20, 22], restriction to female patients [15, 16, 18, 19, 22], and limited types of surgery [15, 16, 18, 19, 21, 22]. Furthermore, most previous studies were based on data that are now more than two decades old [15–19], which can hardly reflect the recent refinements in surgical techniques and anesthetic care (e.g., minimally invasive surgery and multimodal analgesia).

We conducted this single-center, retrospective, matched, cohort study to assess the putative prophylactic effects of droperidol against PONV during the use of morphine-based IV-PCA. We also sought to evaluate the potential effects of droperidol on postoperative sedation, opioid consumption, and pain severity. Based on current evidence [15–22], we hypothesized that the addition of droperidol to IV-PCA infusate was associated with reduced rates and severity of PONV following major surgery.

## Methods

### Clinical setting and patient selection criteria

This study was approved by the Taipei Medical University – Joint Institutional Review Board, Taipei, Taiwan (approval number: TMU-JIRB-N202205095; date of approval: 9 June 2022). The need for written informed consent was waived by the Institutional Review Board due to the retrospective nature of this research. All methods were performed following the standards of the Helsinki Declaration and relevant study guidelines [23].

We used the electronic medical record database of Shuang Ho Hospital, Taipei Medical University to collect retrospective data from 1,512 consecutive patients who underwent surgery with general or neuraxial anesthesia and received opioid-based IV-PCA for postoperative pain control from January 1, 2020 to November 30, 2022. The exclusion criteria were: duplicate cases, missing data of IV-PCA dosage, age < 20 years, using non-morphine analgesics for IV-PCA, and switching to a droperidol regimen during IV-PCA. The included patients were classified into the droperidol group and control group based on whether or not they received the addition of droperidol to IV-PCA. Data were extracted by two independent resident anesthesiologists, who were not involved in the data analysis. The quality of the datasets was validated using random sampling by other authors.

### Intravenous patient-controlled analgesia protocol

Contraindications to IV-PCA were the inability to maintain consciousness, cognitive impairment, and postoperative mechanical ventilation support or intensive care beyond 24 h. IV-PCA was typically initiated at the post-anesthesia care unit after surgery and administered using an ambulatory infusion pump (CADD®-Solis Infusion System, Smiths Medical, Inc., Minneapolis, MN, USA), programmed to deliver morphine sulfate 1 mg/mL in normal saline [24, 25]. The infusion settings were a loading dose of 0–5.0 mL, a demand dose of 0.5–2.0 mL, a basal infusion rate of 0–1.5 mL/hr, and a lockout time of 5–10 min. As an antiemetic prophylaxis, we used a droperidol regimen of 0.025–0.075 mg/mL added to the IV-PCA infusate based on previous literature [15–22, 26, 27]. In the non-droperidol regimen, no antiemetic was added to the morphine solution. The pain service team evaluated the patients’ response at 12-hourly intervals, and more frequently in patients with inadequate analgesia or relevant adverse events (e.g., nausea, vomiting, and sedation). The severity of PONV was rated using a 4-point verbal descriptive scale: no PONV: no complaint of nausea or vomiting; mild PONV: patients complained of nausea but refused antiemetic drugs; moderate PONV: patients complained of nausea and requested antiemetic drugs; severe PONV: patients complained of nausea and had episodes of vomiting requiring antiemetic treatments [20, 28]. For patients with mild PONV, the IV-PCA infusion parameter was adjusted to reduce morphine dosage. When patients had moderate-to-severe PONV, antiemetic medications were administered, and IV-PCA infusion rates were reduced to relieve the symptom. The pharmacologic treatment included dopamine antagonists (e.g., metoclopramide and prochlorperazine), 5-HT3 antagonists (e.g., ondansetron), corticosteroids (e.g., dexamethasone), and histamine antagonists (e.g., diphenhydramine). In most patients, IV-PCA was used for 48 to 72 h and switched to oral acetaminophen or nonsteroidal anti-inflammatory drugs thereafter.

### Study outcomes

The primary outcome was the rate of any episodes of nausea and/or vomiting within 72 h after surgery. The secondary outcomes were the rate of PONV which needed rescue antiemetic medications, the severity of PONV, unintentional sedation within 72 h after surgery, postoperative opioid consumption, and daily maximum pain scores within 72 h after surgery. The occurrence of PONV, level of sedation, and pain intensity were evaluated regularly by certified nurse anesthetists of the pain service team at 12-hourly intervals at the institution. The medical records were reviewed to determine whether the patients received antiemetic medications for PONV during IV-PCA. The University of Michigan Sedation Scale (UMSS) grading system was used to evaluate the sedation level during IV-PCA, as follows: 0, fully awake; 1, drowsy with closed eyes; 2, easily aroused with light tactile stimulation or simple verbal commands; 3, arousable only by strong physical stimulation; 4, unarousable [29]. Unintentional sedation was defined as a maximum UMSS score ≥ 1 after excluding planned sedation. Postoperative pain intensity was evaluated both at rest and during movement using a self-reported 11-point numeric rating scale (NRS) with response options from “no pain” (0) to “the worst pain” (10).

### Anesthesia management

All patients received a 12-lead electrocardiogram before surgery to rule out clinically important QTc prolongation and severe cardiac arrhythmia. General anesthesia was typically induced using fentanyl 1–2 µg/kg and propofol 1–2 mg/kg. Rocuronium 0.6–1.0 mg/kg or cisatracurium 0.1–2.0 mg/kg was given for endotracheal intubation. Inhalational sevoflurane or desflurane was used to maintain general anesthesia. Reversal agents were always administered when neuromuscular blocking agents were used, including sugammadex 2 mg/kg or neostigmine 0.05 mg/kg. For spinal anesthesia, bupivacaine 6–15 mg without opioids was used. In combined general and neuraxial anesthesia, we used a continuous epidural infusion of ropivacaine 5 mg/mL with or without fentanyl 2.5–5 µg/mL. Midazolam 2–5 mg was given intravenously during the induction of general anesthesia or neuraxial anesthesia for anxiolysis on a case-by-case basis. In perioperative fluid management, crystalloid fluids (sodium chloride 0.9% or lactated Ringer’s solution) were administered according to the current practice guidelines [30].

### Covariates for adjustment

To adjust for potential confounding factors, the following patient and clinical covariates were collected based on the available data, physiological plausibility, and existing literature [3, 31]. Demographic attributes were age, sex, and body mass index. The recorded preoperative clinical covariates were the American Society of Anesthesiologists physical status, current cigarette smoking (within 30 days before surgery), previous history of PONV, coexisting diseases (hypertension, diabetes mellitus, major depression, and malignancy), and preoperative blood tests (hemoglobin, creatinine, aspartate aminotransferase, alanine aminotransferase, and estimated glomerular filtration rate based on the Cockcroft-Gault formula) [3, 32]. Intraoperative variables included the site of surgery (categorized into extremity, head and neck, breast, upper abdomen, lower abdomen, thorax, spine, and other), uses of laparoscopic or robotic techniques, types of anesthesia, use of volatile anesthetics, total intravenous anesthesia, duration of anesthesia, intraoperative blood loss and fluid volume, intraoperative use of dexamethasone and midazolam, intraoperative use of non-steroidal anti-inflammatory drugs, intraoperative opioid dosage, and type of neuromuscular blockade reversal agent (neostigmine, sugammadex, or nothing) [3, 31]. Postoperative factors included the duration of IV-PCA, postoperative use of non-steroidal anti-inflammatory drugs, and opioid consumption within 72 h after surgery [3]. The dosages of non-morphine opioids were transformed into morphine milligram equivalents for analysis (Supplementary Table S1) [33, 34].

### Statistical analysis

Normality of the included variables was checked using the Shapiro-Wilk test and Anderson-Darling test. Normally distributed data were expressed as mean ± standard deviation. Non-normally distributed variables were presented as median with inter-quartile range and log-transformed to reduce distribution skewness, including preoperative blood test results, intraoperative blood loss and fluid volume, duration of anesthesia, duration of IV-PCA, and intraoperative and postoperative opioid consumption. To minimize potential confounding effects, a propensity score matching procedure was performed as follows. First, non-parsimonious multivariable logistic regression analyses were used to estimate a propensity score for the patients who did and did not receive droperidol. The patients who received droperidol were matched to those who did not in a ratio of 1:1 using a greedy matching algorithm within a caliper width of 0.05 standard deviations of the log odds of the calculated propensity score and without replacement to adjust for all of the collected covariates. Baseline patient characteristics were compared between the patients who did and did not receive droperidol using the absolute standardized mean difference (ASMD) [35]. Covariate balance between groups was defined as an ASMD less than 0.1 [36]. Conditional logistic regression analyses were used to evaluate associations between the collected variables and PONV. The significant variables in univariate analysis were incorporated into multivariable models to calculate adjusted odds ratios (aORs) with 95% confidence intervals (CIs) for the independent factors of PONV. For sensitivity analysis, the inverse probability treatment weighting (IPTW) method was used to eliminate potential confounding effects of imbalances in the collected covariates, as previously described [37]. Briefly, the inverse of estimated propensity score was used for weighted logistic regression analyses. We truncated 1% of subjects that were at the end of weighting distribution to decrease the effect of the large weights. Subgroup analyses according to Apfel’s simplified risk score (i.e., female sex, non-smoker, history of PONV, and postoperative opioids) [38], age, sex, current smoker or not, previous PONV or not, type of anesthesia, use of volatile anesthetics or not, use of neostigmine or sugammadex, and intraoperative use of dexamethasone or not were also conducted to examine the association of droperidol with PONV in these strata. The aORs were transformed into relative risks using Zhang and Kai’s method [39]. A meta-analysis by Weibel et al. showed that droperidol reduced the risk of PONV by 39% compared to placebo in adults undergoing general anesthesia [13]. For sample size estimation, at least 347 patients in each group were required to detect a relative risk of 0.61 between the droperidol group and control group, accepting a type I error of 5% and type II error of 20% with an anticipated PONV rate of 20% in the control group [13, 40]. The number of patients enrolled in this cohort substantially exceeded the minimum necessary sample size. Furthermore, Austin et al. indicated that at least 20 events per variable are required in logistic regression analyses [41]. The number of PONV events in the matched cohort was 187, suggesting that a maximum of 9 variables could be considered in the multivariable model without affecting the model performance. Since we included 3 variables in the final model, our analyses met the requirement of this criterion. A two-sided *p* value of < 0.05 was used to define a statistically significant difference. All statistical analyses were conducted using SAS software, version 9.4 (SAS Institute Inc., Cary, NC, USA).

## Results

### Baseline patient characteristics

**Fig. 1:**
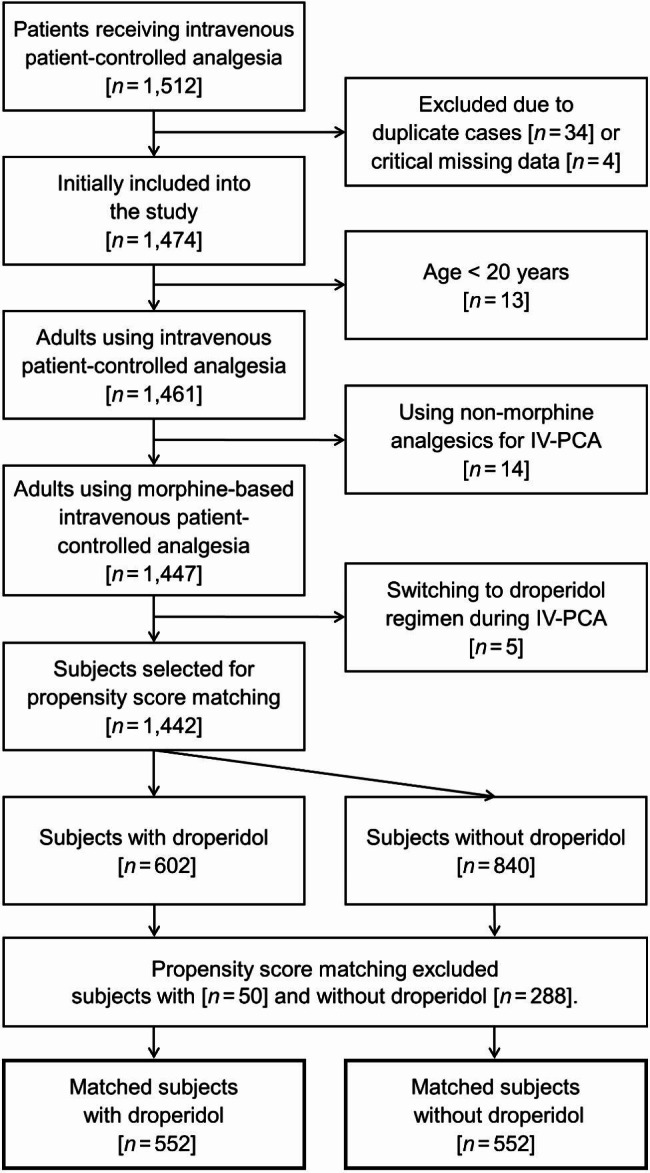
Flow diagram for patient selection

A total of 1,442 patients, 602 in the droperidol group and 840 in the control group, were included before matching. The distribution of baseline patient characters before matching is shown in Supplementary Table S2. After propensity score matching, 552 matched pairs were included for analysis (Fig. 1). All of the baseline patient and clinical characteristics were balanced after matching, except for slightly lower rates of hypertension (23.4% vs. 27.7%) and postoperative uses of non-steroidal anti-inflammatory drugs (3.8% vs. 4.5%) in the droperidol group (Table 1). The median total dose of morphine in IV-PCA was 39.7 mg (interquartile range: 22.4–67.2 mg) in the droperidol group and 39.8 mg (23.0–68.5 mg) in the control group (ASMD = 0.0050). Among the droperidol group, 11 (2.0%), 538 (97.5%), and 2 (0.4%) patients received droperidol at a concentration of 0.025, 0.050, and 0.075 mg/mL, respectively. The cumulative droperidol dose in the droperidol group was 2.0 mg (interquartile range: 1.2–3.4 mg).

**Table 1:** Baseline characteristics of patients with or without receiving droperidol after matching

	Droperidol (*n* = 552)	Control (*n* = 552)	ASMD
**Age, year**	48.7 ± 14.9	49.6 ± 15.9	0.0584
**Sex, male**	85 (15.4%)	97 (17.6%)	0.0872
**Body mass index, kg/m** ^**2**^	26.0 ± 4.8	26.1 ± 4.7	0.0211
**ASA class**			0.0047
I	92 (16.7%)	91 (16.5%)	
II	456 (82.6%)	459 (83.2%)	
III	4 (0.7%)	2 (0.4%)	
**Apfel’s risk score**	2.8 ± 0.6	2.8 ± 0.6	0.0185
**Current smoker**	55 (10.0%)	49 (8.9%)	0.0703
**Previous PONV**	25 (4.5%)	25 (4.5%)	< 0.0001
**Hypertension**	129 (23.4%)	153 (27.7%)	0.1263
**Diabetes mellitus**	75 (13.6%)	84 (15.2%)	0.0730
**Major depression**	9 (1.6%)	8 (1.5%)	0.0660
**Malignancy**	83 (15.0%)	78 (14.1%)	0.0401
**Preoperative blood test**			
Hemoglobin, g/dL	12.6 (11.3–13.7)	12.5 (11.4–13.7)	0.0157
eGFR, mL/min/1.73 m^2^	97.2 (81.6–111.0)	96.1 (79.3–113.2)	0.0273
Alanine aminotransferase, U/L	18 (13–26)	18 (14–27)	0.0687
Aspartate aminotransferase, U/L	20 (16–26)	20 (17–26)	0.0335
**Surgical site**			0.0174
Extremity	101 (18.3%)	106 (19.2%)	
Head and neck	7 (1.3%)	10 (1.8%)	
Breast	11 (2.0%)	10 (1.8%)	
Upper abdomen	44 (8.0%)	46 (8.3%)	
Lower abdomen	342 (62.0%)	327 (59.2%)	
Thorax	7 (1.3%)	8 (1.5%)	
Spine	34 (6.2%)	38 (6.9%)	
Other^†^	6 (1.1%)	7 (1.3%)	
**Laparoscopic or robotic surgery**	79 (14.3%)	76 (13.8%)	0.0248
**Intraoperative blood loss, mL**	200 (10–550)	200 (10–530)	0.0302
**Type of anesthesia**			0.0499
Neuraxial anesthesia	184 (33.3%)	172 (31.2%)	
General anesthesia	367 (66.5%)	378 (68.5%)	
Combined general and neuraxial anesthesia	1 (0.2%)	2 (0.4%)	
**Use of volatile anesthetics**	368 (66.7%)	380 (68.8%)	0.0549
**Anesthesia duration, min**	155 (105–230)	155 (105–238)	0.0273
**Intraoperative fluid volume, mL**	900 (650–1200)	900 (650–1200)	0.0094
**Intraoperative use of dexamethasone**	345 (62.5%)	357 (64.7%)	0.0518
**Intraoperative use of midazolam**	99 (17.9%)	90 (16.3%)	0.0634
**Intraoperative use of NSAIDs**	89 (16.1%)	79 (14.3%)	0.0775
**Intraoperative opioid consumption, MME**	10.0 (3.3–15.0)	10.0 (3.3–15.0)	0.0175
**Neuromuscular blockade reversal agent**			0.0187
Nil	188 (34.1%)	178 (32.3%)	
Neostigmine	123 (22.3%)	134 (24.3%)	
Sugammadex	241 (43.7%)	240 (43.5%)	
**PCA duration, hour**	69.4 (64.0–72.7)	68.6 (63.5–72.3)	0.0144
**Postoperative use of NSAIDs**	21 (3.8%)	25 (4.5%)	0.1003
**Postoperative opioid consumption, MME**	39.8 (23.0–68.8)	39.7 (22.2–67.4)	0.0099

Values were mean ± standard deviation, median (interquartile range) or counts (percent). ASA = American Society of Anesthesiologists; ASMD = absolute standardized mean difference; eGFR = estimated glomerular filtration rate; MME = morphine milligram equivalent; NSAIDs = non-steroidal anti-inflammatory drugs; PONV = postoperative nausea and vomiting. † Includes anal surgeries, hernia repair, and surgeries involving multiple sites

### Postoperative nausea and vomiting

**Fig. 2:**
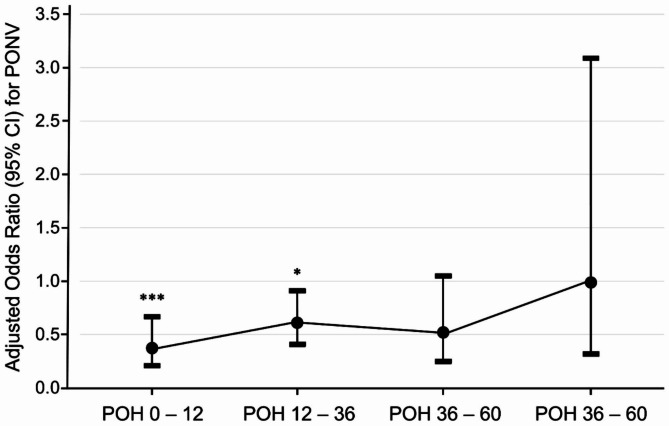
Antiemetic effect of droperidol attenuated 36 h after surgery (*** *p* < 0.001, *p < 0.05; CI = confidence interval, POH = postoperative hour, PONV = postoperative nausea and vomiting)

A total of 67 patients (12.1%) in the droperidol group developed PONV compared to 120 (21.7%) in the control group, and the absolute risk reduction was 9.6% (95% CI: 5.2–14.0, *p* < 0.0001). Table 2 shows the results of univariate and multivariable analyses for PONV. After adjusting for covariates, droperidol was significantly associated with a reduction in PONV (aOR: 0.49, 95% CI: 0.35–0.67, *p* < 0.0001; relative risk: 0.55, 95% CI: 0.41–0.72). Other independent factors for PONV were sex (male vs. female, aOR: 0.51, 95% CI: 0.30–0.85, *p* = 0.0101), and previous history of PONV (aOR: 2.74, 95% CI: 1.48–5.05, *p* = 0.0013). The reduction in PONV risk after droperidol prophylaxis was confirmed using the IPTW method (aOR: 0.60, 95% CI: 0.49–0.72, *p* < 0.0001). The rate and severity of PONV within 72 h after surgery are shown in Table 3. Of note, the protective effect of droperidol was significant within 36 h after surgery and attenuated thereafter (Fig. 2). Furthermore, the patients who used droperidol received fewer rescue antiemetics for PONV (aOR: 0.58, 95% CI: 0.41–0.80, *p* = 0.0011).

**Table 2:** Univariate and multivariable analyses for postoperative nausea and vomiting

	Univariate cOR (95% CI)	*p*	Multivariable aOR (95% CI)	*p*
**Droperidol vs. control**	0.50 (0.36–0.69)	0.0046	0.49 (0.35–0.67)	< 0.0001
**Age, year**	0.995 (0.985–1.005)	0.3339	.	.
**Sex, male**	0.49 (0.29–0.82)	0.0064	0.51 (0.30–0.85)	0.0101
**Body mass index, kg/m** ^**2**^	0.99 (0.95–1.02)	0.4442	.	.
**ASA class**		0.6891	.	.
II vs. I	0.84 (0.56–1.26)	0.8651	.	.
III vs. I	0.85 (0.10–7.47)	0.9436	.	.
**Current smoker**	0.88 (0.51–1.54)	0.6573	.	.
**Previous PONV**	2.95 (1.62–5.37)	0.0004	2.74 (1.48–5.05)	0.0013
**Hypertension**	0.68 (0.46–1.00)	0.0488	.	.
**Diabetes mellitus**	0.95 (0.61–1.50)	0.8313	.	.
**Major depression**	0.65 (0.15–2.87)	0.5704	.	.
**Malignancy**	0.79 (0.49–1.27)	0.3326	.	.
**Preoperative blood test** ^†^			.	.
Hemoglobin, g/dL	0.84 (0.43–1.64)	0.6098	.	.
eGFR, mL/min/1.73 m^2^	1.48 (1.00–2.19)	0.0502	.	.
Alanine aminotransferase, U/L	0.89 (0.70–1.12)	0.3126	.	.
Aspartate aminotransferase, U/L	1.02 (0.77–1.35)	0.8753	.	.
**Surgical site, extremity as reference**		0.0597	.	.
Head and neck	0.26 (0.03–2.03)	0.2675	.	.
Breast	0.44 (0.10–1.96)	0.4636	.	.
Upper abdomen	0.58 (0.28–1.19)	0.5329	.	.
Lower abdomen	0.90 (0.61–1.35)	0.3069	.	.
Thorax	2.09 (0.68–6.45)	0.0393	.	.
Spine	0.31 (0.12–0.82)	0.0624	.	.
Other	1.86 (0.54–6.33)	0.0905	.	.
**Laparoscopic or robotic surgery**	0.83 (0.52–1.34)	0.4528	.	.
**Intraoperative blood loss, mL** ^†^	1.03 (0.96–1.10)	0.3935	.	.
**Type of anesthesia**		0.4611	.	.
General vs. neuraxial anesthesia	0.84 (0.61–1.18)	0.3666	.	.
Combined vs. neuraxial anesthesia	2.20 (0.20–24.59)	0.4775	.	.
**Use of volatile anesthetics**	0.85 (0.61–1.18)	0.3282	.	.
**Anesthesia duration, min** ^†^	0.88 (0.71–1.08)	0.2128	.	.
**Intraoperative fluid volume, mL** ^†^	0.94 (0.77–1.16)	0.5844	.	.
**Intraoperative use of dexamethasone**	0.85 (0.62–1.17)	0.3249	.	.
**Intraoperative use of midazolam**	1.19 (0.80–1.78)	0.3962	.	.
**Intraoperative use of NSAIDs**	1.30 (0.86–1.97)	0.2164		
**Intraoperative opioid consumption, MME** ^†^	0.87 (0.71–1.06)	0.1705	.	.
**Neuromuscular blockade reversal**		0.5673	.	.
Neostigmine vs. nil	0.81 (0.53–1.24)	0.4762	.	.
Sugammadex vs. nil	0.86 (0.60–1.23)	0.7952	.	.
**IV-PCA duration, hour** ^†^	1.13 (0.79–1.60)	0.5016	.	.
**Postoperative use of NSAIDs**	0.46 (0.16–1.29)	0.1374		
**Postoperative opioid consumption, MME** ^†^	0.89 (0.79–1.00)	0.0547	.	.

aOR, adjusted odds ratio; ASA = American Society of Anesthesiologists; CI = confidence interval; cOR = crude odds ratio; eGFR = estimated glomerular filtration rate; IV-PCA = intravenous patient-controlled analgesia; MME = morphine milligram equivalent; NSAIDs = non-steroidal anti-inflammatory drugs; PONV = postoperative nausea and vomiting. † On base-2 logarithmic scale

**Table 3:** Rates and severity of postoperative nausea and vomiting among patients with or without receiving droperidol

	Droperidol	Control	cOR (95% CI)	*p*	aOR (95% CI)^†^	*p*
	Event, *n* (%)	Event, *n* (%)				
**All PONV**	67 (12.1%)	120 (21.7%)	0.50 (0.36–0.69)	0.0046	0.49 (0.35–0.67)	< 0.0001
POH 0–12	17 (3.1%)	42 (7.6%)	0.39 (0.22–0.69)	0.0012	0.37 (0.21–0.67)	0.0009
Mild	13 (2.4%)	28 (5.1%)				
Moderate	3 (0.5%)	9 (1.6%)				
Severe	1 (0.2%)	5 (0.9%)				
POH 12–36	45 (8.2%)	69 (12.5%)	0.62 (0.42–0.92)	0.0184	0.61 (0.41–0.91)	0.0151
Mild	32 (5.8%)	50 (9.1%)				
Moderate	9 (1.6%)	17 (3.1%)				
Severe	4 (0.7%)	2 (0.4%)				
POH 36–60	12 (2.2%)	23 (4.2%)	0.51 (0.25–1.04)	0.0632	0.52 (0.25–1.05)	0.0673
Mild	7 (1.3%)	21 (3.8%)				
Moderate	3 (0.5%)	2 (0.4%)				
Severe	2 (0.4%)	0 (0)				
POH 60–72	6 (1.1%)	6 (1.1%)	1.00 (0.32–3.13)	0.9949	0.99 (0.32–3.09)	0.9799
Mild	6 (1.1%)	5 (0.9%)				
Moderate	0 (0)	1 (0.2%)				
Severe	0 (0)	0 (0)				
**Need for antiemetics**	71 (12.9%)	110 (19.9%)	0.59 (0.43–0.82)	0.0016	0.58 (0.41–0.80)	0.0011

aOR, adjusted odds ratio; CI, confidence interval; cOR, crude odds ratio; POH, postoperative hour

† Adjusted for sex and previous history of PONV.

### Subgroup analyses

Droperidol was associated with a decreased risk of PONV in the subgroups of Apfel’s score = 1 or 2 (aOR: 0.36), Apfel’s score = 3 or 4 (aOR: 0.51), age < 65 years (aOR: 0.47), female sex (aOR: 0.49), not a current smoker (aOR: 0.51), no history of PONV (aOR: 0.47), neuraxial anesthesia (aOR: 0.54), general anesthesia (aOR: 0.45), use of volatile anesthetics (aOR: 0.45), no use of volatile anesthetics (aOR:0.54), use of sugammadex (aOR: 0.42), intraoperative use of dexamethasone (aOR: 0.46), and no intraoperative use of dexamethasone (aOR: 0.51) (Table 4).

**Table 4:** Subgroup analyses for postoperative nausea and vomiting

Subgroup	Droperidol or control	Event, *n* (%)	cOR (95% CI)	*p*	aOR (95% CI)^†^	*p*
Apfel’s score = 1 or 2	Droperidol	8 (7.3%)	0.40 (0.17–0.96)	0.0396	0.36 (0.15–0.89)	0.0258
	Control	19 (16.4%)	reference		reference	
Apfel’s score = 3 or 4	Droperidol	59 (13.4%)	0.51 (0.36–0.73)	0.0002	0.51 (0.36–0.72)	0.0002
	Control	101 (23.2%)	reference		reference	
Age < 65 years	Droperidol	57 (12.5%)	0.47 (0.33–0.67)	< 0.0001	0.47 (0.33–0.67)	< 0.0001
	Control	102 (23.4%)	reference		reference	
Age ≥ 65 years	Droperidol	10 (10.3%)	0.63 (0.27–1.43)	0.2657	0.56 (0.24–1.30)	0.1766
	Control	18 (15.5%)	reference		reference	
Male	Droperidol	5 (5.9%)	0.40 (0.14–1.18)	0.0986	0.40 (0.14–1.18)	0.0986
	Control	13 (13.4%)	reference		reference	
Female	Droperidol	62 (13.3%)	0.50 (0.35–0.70)	< 0.0001	0.49 (0.35–0.70)	< 0.0001
	Control	107 (23.5%)	reference		reference	
Current smoker	Droperidol	4 (7.3%)	0.24 (0.07–0.81)	0.0213	0.29 (0.08–1.03)	0.0558
	Control	12 (24.5%)	reference		reference	
Not a current smoker	Droperidol	63 (12.7%)	0.53 (0.38–0.75)	0.0003	0.51 (0.37–0.72)	0.0001
	Control	108 (21.5%)	reference		reference	
Previous PONV	Droperidol	8 (32.0%)	0.71 (0.22–2.25)	0.5563	0.71 (0.22–2.3)	0.5563
	Control	10 (40.0%)	reference		reference	
No previous PONV	Droperidol	59 (11.2%)	0.48 (0.34–0.67)	< 0.0001	0.47 (0.33–0.66)	< 0.0001
	Control	110 (20.9%)	reference		reference	
Neuraxial anesthesia	Droperidol	26 (14.1%)	0.54 (0.32–0.94)	0.0282	0.54 (0.32–0.94)	0.0286
	Control	40 (23.3%)	reference		reference	
General anesthesia	Droperidol	41 (11.2%)	0.48 (0.32–0.72)	0.0004	0.45 (0.30–0.69)	0.0002
	Control	79 (20.9%)	reference		reference	
Use of volatile anesthetics	Droperidol	41 (11.1%)	0.47 (0.31–0.71)	0.0003	0.45 (0.30–0.68)	0.0002
	Control	80 (21.1%)	reference		reference	
No use of volatile anesthetics	Droperidol	26 (14.1%)	0.54 (0.32–0.94)	0.0282	0.54 (0.32–0.94)	0.0286
	Control	40 (23.3%)	reference		reference	
Use of neostigmine	Droperidol	13 (10.6%)	0.47 (0.23–0.96)	0.0371	0.49 (0.24–1.02)	0.0569
	Control	27 (20.2%)	reference		reference	
Use of sugammadex	Droperidol	27 (11.2%)	0.46 (0.28–0.76)	0.0023	0.42 (0.25–0.70)	0.0009
	Control	52 (21.7%)	reference		reference	
Intraoperative use of dexamethasone	Droperidol	39 (11.3%)	0.49 (0.32–0.74)	0.0008	0.46 (0.30–0.71)	0.0004
	Control	74 (20.7%)	reference		reference	
No intraoperative use of dexamethasone	Droperidol	28 (13.5%)	0.51 (0.30–0.85)	0.0100	0.51 (0.30–0.85)	0.0101
	Control	46 (23.6%)	reference		reference	

aOR, adjusted odds ratio; CI, confidence interval; cOR, crude odds ratio; PONV, postoperative nausea and vomiting

† Adjusted for sex and previous history of PONV.

### Unintentional sedation, opioid consumption, and pain intensity

The rate of unintentional sedation was comparable between the droperidol group (9.1%) and control group (7.8%; *p* = 0.4481) (Table 5). Postoperative opioid consumption and daily maximum NRS pain scores were also similar between the two groups.

**Table 5:** Unintentional sedation, postoperative opioid consumption, and pain intensity among patients with or without droperidol

	Droperidol (*n* = 552)	Control (*n* = 552)	*p*
**Unintentional sedation**	50 (9.1%)	43 (7.8%)	0.4481
**Maximum UMSS score** ^**†**^			0.3981
0	502 (90.9%)	509 (92.2%)	
1	22 (4.0%)	23 (4.2%)	
2	23 (4.2%)	19 (3.4%)	
3	5 (0.9%)	1 (0.2%)	
4	0 (0)	0 (0)	
**Postoperative opioid consumption, MME**	39.8 (23.0–68.8)	39.7 (22.2–67.4)	0.5271
**Mean daily maximum NRS pain score**	2.9 (2.4–3.5)	3.0 (2.4–3.5)	0.7660
**Daily maximum NRS pain score**			
POH 0–12, at rest	3.0 (2.0–4.0)	3.0 (2.0–4.0)	0.7251
POH 0–12, during movement	5.0 (4.0–6.0)	5.0 (4.0–6.0)	0.6789
POH 12–36, at rest	2.0 (1.0–3.0)	2.0 (2.0–3.0)	0.2208
POH 12–36, during movement	4.0 (3.0–5.0)	4.0 (3.0–6.0)	0.4338
POH 36–60, at rest	2.0 (1.0–2.0)	2.0 (1.0–2.0)	0.8051
POH 36–60, during movement	3.0 (3.0–5.0)	3.0 (3.0–4.0)	0.8522
POH 60–72, at rest	1.0 (0–2.0)	1.0 (0–2.0)	0.4523
POH 60–72, during movement	2.0 (2.0–3.0)	2.0 (2.0–3.0)	0.5807

Values were counts (percent) or median (interquartile range). MME = morphine milligram equivalent; NRS = numeric rating scale; POH, postoperative hour; UMSS = The University of Michigan Sedation Scale

† 0, fully awake; 1, drowsy with closed eyes; 2, easily aroused with light tactile stimulation or simple verbal commands; 3, arousable only by strong physical stimulation; 4, unarousable

## Discussion

In this matched cohort study, we found that the addition of droperidol was significantly associated with a reduced rate of PONV in morphine-based IV-PCA. Subgroup analyses showed that the prophylactic effect of droperidol was significant among the patients who were younger than 65 years, female, not a current smoker, and did not have a history of PONV. Furthermore, the patients who received droperidol required fewer rescue antiemetics for PONV compared to their counterparts. Noticeably, no additional risk of unintentional sedation related to droperidol was observed in this study. Our results provide important evidence to support the clinical benefits of droperidol in antiemetic prophylaxis during intravenous opioid analgesia.

The antiemetic efficacy of droperidol in opioid-based IV-PCA has been evaluated in previous studies [15–22, 26, 27]. However, most studies have included a small sample size [15–22, 26, 27], which may have reduced the statistical power and limited the inclusion and adjustment for various covariates. Given that the cause of PONV is multifactorial, our matching analyses were based on a large patient sample and carefully controlled patient-related, surgery-related, and anesthesia-related parameters. This reflected an actual clinical setting and minimized potential confounding effects. In addition, some prior studies focused on female patients [15, 16, 18, 19, 22, 26], gynecological surgeries [15, 16, 18, 19, 22, 26], and non-abdominal surgeries [21], thereby limiting the external validity of the study results. Our findings suggested that the addition of droperidol to IV-PCA effectively decreased the rate of PONV within 36 h after surgery, but that the antiemetic effect was attenuated thereafter. This finding is similar to most previous studies [15–19, 21, 22] but in contrast to another [20]. In that study, Kuo et al. reported that the addition of droperidol significantly reduced the incidence and severity of PONV on postoperative days 2 and 3, but not on day 1 [20]. This discrepancy may be due to variations in the patients’ baseline risk of PONV, IV-PCA infusion settings, timing of evaluations, and diagnostic methods and definitions. In our study, the attenuated protective effect of droperidol against PONV may be explained by differences in the mechanisms between early and late PONV. Late PONV may be partly caused by opioid-related gastroparesis and ileus, for which droperidol is not an effective treatment [42]. Our results suggest that additional measures are needed to prevent late PONV, such as pharmacologic combination therapies [4]. Our analyses demonstrated a decreased risk of PONV in the subgroups of age < 65 years, female sex, not a current smoker, and no history of PONV. Current practice guidelines state that antiemetic prophylaxis is more effective in high-risk patients [4]. Our non-statistically significant results in some subgroups (i.e., age ≥ 65 years, male sex, current smokers, previous PONV, and use of neostigmine) should be interpreted with caution due to the small sample size and possibility of underpowered statistics. Future studies are required to evaluate the antiemetic efficacy of droperidol in low-risk patients while carefully controlling for patient and clinical factors. Current smoking is an established protective factor for PONV [38, 43]. Our analysis demonstrated a lower rate of PONV in the current smokers who used droperidol compared to the controls, although without statistical significance. The exact mechanism of how smoking reduces PONV is not fully understood. One potential explanation is the presence of antiemetic substances in tobacco smoke, which act at the pharmacological receptors (e.g., D2 receptors and 5-HT3 receptors) mediating PONV [44]. Another possible explanation is that chronic smoking may up-regulate the activity of hepatocellular enzymes (e.g., cytochrome P450) and enhance the metabolism of emetic anesthetic drugs [44]. Further studies are needed to clarify the interaction between droperidol and modified D2 receptors in chronic smokers.

Noticeably, we found no difference in sedation level between the droperidol and control groups in those who received droperidol 0.025–0.075 mg/mL added to the morphine solution. Since both droperidol and morphine may induce sedation, it is crucial to determine the effective and safe concentration of droperidol when used with opioids in terms of antiemesis and sedation. Previous studies have reported that droperidol 0.017–0.10 mg/mL added to IV-PCA infusate did not increase the risk of sedation or consciousness disturbance after surgery [16, 18, 19]. However, a droperidol regimen of 0.125–0.20 mg/mL has been reported to cause sedation or drowsiness during IV-PCA [15, 17, 19]. Lamon et al. compared four droperidol regimens added to IV-PCA and found that a dose of 0.10 mg/mL appeared to be optimal with regards to the antiemetic efficacy and sedation risk [19]. Taken together, these and our findings suggest that the addition of droperidol 0.025–0.10 mg/mL to opioid-based IV-PCA seems to be appropriate from a benefit-risk standpoint. We also found that among the patients with a Apfel’s score of 3 or 4, the addition of droperidol reduced the rate of PONV to 13.4% compared to 23.2% in the control group. Current practice guidelines recommend the use of combination antiemetic therapy and multimodal systemic analgesia for patients at high risk of PONV [4]. Droperidol combined with 5-HT3 receptor antagonists has been demonstrated to be superior compared to single drugs in preventing PONV [4]. Furthermore, the addition of acetaminophen, nonsteroidal anti-inflammatory drugs, and ketamine has been shown to be efficacious in decreasing the use of opioids and related adverse effects [4].

In December 2001, the United States Food and Drug Administration issued a black box warning indicating an association between droperidol, QT prolongation, and torsades de pointes, although high-quality clinical trials did not support this hypothesis [2, 4, 8, 13, 14]. Consequently, droperidol was rapidly replaced by 5-HT3 receptor antagonists, neurokinin blockers, and atypical antipsychotics. Experts argued that the warning about droperidol was primarily based on nine case reports without solid evidence of causation [45]. Several randomized controlled trials demonstrated that the QT prolongation related to droperidol was transient, dose-dependent and did not develop into serious ventricular arrhythmias or cardiac arrest at therapeutic doses (0.625–1.25 mg) [13]. In 2015, the American Academy of Emergency Medicine stated that the evidence supporting the black box warning was inadequate, and supported the use of droperidol in an emergency setting [46]. In February 2019, droperidol was reintroduced to the United States drug market by American Regent, Inc. (Shirley, NY, USA), still with the black box warning. To date, no study has investigated the changes in baseline rates of PONV before and after the black box warning. Further studies and real-world data are needed to evaluate the actual impact of the reintroduction of droperidol on the incidence of PONV.

There are several limitations to this study. First, although propensity score matching was used to reduce the imbalance in baseline patient characteristics between groups, unmeasured characteristics and confounders (e.g., anxiety and menstrual cycle) could not further controlled [4]. In addition, the outcome assessors were not blinded, and perioperative care was not standardized due to the retrospective design of this study. Second, the IV-PCA infusion settings varied considerably, and this may have led to a wide variation in the dosing of morphine and droperidol and confounded the risk of PONV and sedation. Well-designed prospective studies are warranted to clearly elucidate the clinical benefits and safety of droperidol in antiemesis during systemic opioid analgesia. Third, nausea and vomiting were not assessed separately, and thus we could not analyze the distinct effects of droperidol on these two outcomes. Fourth, we did not adjust for 5-HT3 receptor antagonists, neurokinin antagonists, or a scopolamine patch because these agents are rarely used for antiemetic prophylaxis for surgical patients in our hospital. Fifth, some potential adverse effects related to droperidol were not evaluated due to a lack of complete data, such as QT prolongation, transient ventricular arrhythmia, and extrapyramidal symptoms [47].

## Conclusions

The addition of droperidol to morphine-based IV-PCA was associated with lower rates of PONV and the need for antiemetic medications. No additional risk of unintentional sedation attributable to droperidol was found in this single-center matched cohort. The antiemetic benefit of droperidol was especially significant within 36 h after surgery and attenuated thereafter. Our results suggest that the addition of droperidol to IV-PCA is effective in the prophylaxis of PONV, and that it does not affect sedation risk, opioid requirements, or pain intensity. We suggest the following issues for future studies: the prophylactic role of droperidol against PONV in low-risk patients, the optimal concentration of droperidol in morphine-based IV-PCA in terms of antiemesis and sedation, and the efficacy and cost of combination antiemetic regimens compared to droperidol alone. Lastly, droperidol should be used cautiously in patients with preexisting QT prolongation or the concomitant use of drugs that potentially prolong the QT interval.

### Electronic supplementary material

Below is the link to the electronic supplementary material.

Supplementary Material 1

## Data Availability

The data presented in this study are available on request from the corresponding author. The data are not publicly available due to the regulations of the Institutional Review Board.

## Abbreviations

5-HT3: 5-hydroxytryptamine 3
aOR: Adjusted odds ratio
ASA: American Society of Anesthesiologists
ASMD: Absolute standardized mean difference
CI: Confidence interval
cOR: Crude odds ratio
eGFR: Estimated glomerular filtration rate
IPTW: inverse probability treatment weighting
IV-PCA: Intravenous patient-controlled analgesia
MME: Morphine milligram equivalent
NRS: Numeric rating scale
POH: Postoperative hour
PONV: Postoperative nausea and vomiting
