# Supplementary material for: Antiemetic prophylaxis with droperidol in morphine-based intravenous patient-controlled analgesia: a propensity score matched cohort study
Source: BMC Anesthesiol. 2023 Oct 28;23:351. doi: 10.1186/s12871-023-02319-2 (PMC10612161; doi:10.1186/s12871-023-02319-2)
Supplement: Supplementary file 1 — Supplementary Material 1 [file 12871_2023_2319_MOESM1_ESM.docx]

Supplementary Table S1: Opioid equianalgesic conversion chart

| **Opioid** | **Administration route** | **Dose equivalent to 1 mg of parenteral morphine (mg)** |
| --- | --- | --- |
| Morphine | Oral | 3 |
| Fentanyl | Intravenous | 0.01 |
| Alfentanil | Intravenous | 0.087 |
| Remifentanil | Intravenous | 0.01 |
| Tramadol | Intravenous | 7.5 |
| Tramadol | Oral | 15 |
| Meperidine | Intravenous | 7.5 |

Supplementary Table S2: Baseline characteristics of patients with or without receiving droperidol before matching

|  | **Droperidol**  **(*n* = 602)** | **Control**  **(*n* = 840)** | **ASMD** |
| --- | --- | --- | --- |
| **Age, year** | 48.7 ± 14.7 | 50.8 ± 16.4 | 0.1336 |
| **Sex, male** | 91 (15.1%) | 190 (22.6%) | 0.2732 |
| **Body mass index, kg/m^2^** | 26.1 ± 5.0 | 26.0 ± 4.8 | 0.0205 |
| **ASA class** |  |  | 0.1568 |
| I | 102 (16.9%) | 113 (13.5%) |  |
| II | 496 (82.4%) | 699 (83.2%) |  |
| III | 4 (0.7%) | 28 (3.3%) |  |
| **Apfel’s risk score** | 2.8 ± 0.6 | 2.7 ± 0.7 | 0.2049 |
| **Current smoker** | 58 (9.6%) | 116 (13.8%) | 0.2246 |
| **Previous PONV** | 30 (5.0%) | 29 (3.5%) | 0.2112 |
| **Hypertension** | 141 (23.4%) | 241 (28.7%) | 0.1512 |
| **Diabetes mellitus** | 80 (13.3%) | 166 (19.8%) | 0.2615 |
| **Major depression** | 10 (1.7%) | 12 (1.4%) | 0.0845 |
| **Malignancy** | 100 (16.6%) | 120 (14.3%) | 0.0983 |
| **Preoperative blood test** |  |  |  |
| Hemoglobin, g/dL | 12.7 (11.3 – 13.7) | 12.4 (11.1 – 13.6) | 0.0510 |
| eGFR, mL/min/1.73 m^2^ | 97.4 (81.9 – 110.8) | 95.4 (78.2 – 111.3) | 0.0685 |
| Alanine aminotransferase, U/L | 18 (13 – 26) | 19 (14 – 28) | 0.1522 |
| Aspartate aminotransferase, U/L | 20 (16 – 26) | 21 (17 – 26) | 0.0922 |
| **Surgical site** |  |  | 0.0576 |
| Extremity | 112 (18.6%) | 185 (22.0%) |  |
| Head and neck | 10 (1.7%) | 10 (1.2%) |  |
| Breast | 15 (2.5%) | 10 (1.2%) |  |
| Upper abdomen | 45 (7.5%) | 96 (11.4%) |  |
| Lower abdomen | 369 (61.3%) | 456 (54.3%) |  |
| Thorax | 9 (1.5%) | 13 (1.6%) |  |
| Spine | 34 (5.7%) | 56 (6.7%) |  |
| Other^†^ | 8 (1.3%) | 14 (1.7%) |  |
| **Laparoscopic or robotic surgery** | 84 (14.0%) | 106 (12.6%) | 0.0639 |
| **Intraoperative blood loss, mL** | 200 (10 – 500) | 200 (10 – 500) | 0.0123 |
| **Type of anesthesia** |  |  | 0.0666 |
| Neuraxial anesthesia | 188 (31.2%) | 280 (33.3%) |  |
| General anesthesia | 406 (67.4%) | 558 (66.4%) |  |
| Combined general and neuraxial anesthesia | 8 (1.3%) | 2 (0.2%) |  |
| **Use of volatile anesthetics** | 414 (68.8%) | 557 (66.3%) | 0.0619 |
| **Anesthesia duration, min** | 160 (105 – 240) | 150 (100 – 230) | 0.0828 |
| **Intraoperative fluid volume, mL** | 900 (650 – 1250) | 900 (600 – 1200) | 0.0937 |
| **Intraoperative use of dexamethasone** | 389 (64.6%) | 523 (62.3%) | 0.0560 |
| **Intraoperative use of midazolam** | 110 (18.3%) | 152 (18.1%) | 0.0066 |
| **Intraoperative use of NSAIDs** | 101 (16.8%) | 101 (12.0%) | 0.2143 |
| **Intraoperative opioid consumption, MME** | 10.0 (3.3 – 15.0) | 10.0 (3.3 – 15.0) | 0.0183 |
| **Neuromuscular blockade reversal agent** |  |  | 0.0635 |
| Nil | 193 (32.1%) | 300 (35.7%) |  |
| Neostigmine | 138 (22.9%) | 178 (21.2%) |  |
| Sugammadex | 271 (45.0%) | 362 (43.1%) |  |
| **PCA duration, hour** | 69.4 (63.9 – 72.5) | 69.1 (63.5 – 72.4) | 0.0512 |
| **Postoperative use of NSAIDs** | 22 (3.7%) | 40 (4.8%) | 0.1523 |
| **Postoperative opioid consumption, MME** | 40.0 (23.0 – 68.0) | 40.5 (21.3 – 69.0) | 0.0167 |

Values were mean ± standard deviation, median (interquartile range) or counts (percent). ASA=American Society of Anesthesiologists; ASMD=absolute standardized mean difference; eGFR=estimated glomerular filtration rate; MME=morphine milligram equivalent; NSAIDs=non-steroidal anti-inflammatory drugs; PONV=postoperative nausea and vomiting. † Includes anal surgeries, hernia repair, and surgeries involving multiple sites.
